# Language-independent talker-specificity in first-language and second-language speech production by bilingual talkers: L1 speaking rate predicts L2 speaking rate

**DOI:** 10.1121/1.4976044

**Published:** 2017-02-16

**Authors:** Ann R. Bradlow, Midam Kim, Michael Blasingame

**Affiliations:** Department of Linguistics, 2016 Sheridan Road, Evanston, Illinois 60208, USA

## Abstract

Second-language (L2) speech is consistently slower than first-language (L1) speech, and L1 speaking rate varies within- and across-talkers depending on many individual, situational, linguistic, and sociolinguistic factors. It is asked whether speaking rate is also determined by a language-independent talker-specific trait such that, across a group of bilinguals, L1 speaking rate significantly predicts L2 speaking rate. Two measurements of speaking rate were automatically extracted from recordings of read and spontaneous speech by English monolinguals (*n* = 27) and bilinguals from ten L1 backgrounds (*n* = 86): speech rate (syllables/second), and articulation rate (syllables/second excluding silent pauses). Replicating prior work, L2 speaking rates were significantly slower than L1 speaking rates both across-groups (monolinguals' L1 English vs bilinguals' L2 English), and across L1 and L2 within bilinguals. Critically, within the bilingual group, L1 speaking rate significantly predicted L2 speaking rate, suggesting that a significant portion of inter-talker variation in L2 speech is derived from inter-talker variation in L1 speech, and that individual variability in L2 spoken language production may be best understood within the context of individual variability in L1 spoken language production.

## I. INTRODUCTION

Cross-language and cross-talker variability are salient features of human speech production. Cross-language variability at the phonetic level is evident in the observation that similar speech sounds in different languages (i.e., speech sounds that are represented by the same symbol in the International Phonetic Alphabet) often vary in their articulatory and acoustic details, giving rise to language-specific or dialect-specific articulatory settings (Honikman, 1964; Laver, 1978; Mennen *et al.*, 2010, and references therein). For example, Bradlow (1995) demonstrated across-the-board fronting (higher second formant frequencies) of English /i, e, o, u/ relative to their Spanish counterparts. Similarly, Recasens (2010) demonstrated dialect-specific constriction anteriority for several front lingual consonants, /t, n, l, s, r, ʧ, ʃ, ʎ, ɲ/, among Catalan dialects. In each of these cases, the documented language- or dialect-dependent differences in articulatory setting prevailed over talker-specific differences. Specifically, the fronted articulatory setting for English relative to Spanish vowels could not be attributed to cross-talker variation in vocal tract length, which would have resulted in parallel shifts for all formant frequencies rather than just for the second formant (Bradlow, 1995). Similarly, for the consonant study (Recasens, 2010) the dialect-specific articulatory setting could not be attributed to individual variation in palate morphology. Instead, studies such as these have demonstrated a group-level, learned, articulatory setting, or language/dialect-specific phonetics.

Within the language/dialect-specific phonetic setting for a given language or dialect, acoustic-phonetic variation across individual talkers can exceed the extent expected based solely on physical variation across individuals. For example, within Standard American English, individual talkers differ in their characteristic voice onset times (VOTs) for voiceless stops. That is, controlling for speaking rate, some talkers produce longer VOTs than other talkers, and these talker differences are consistent across various places of articulation (Allen *et al.*, 2003; Theodore *et al.*, 2009). Within-language talker-specificity in fricative production has also been demonstrated, specifically, with respect to degree of cross-token variability with some talkers showing considerably more within-category variability than others (Newman *et al.*, 2001). Thus, in addition to acquired articulatory settings at the group level (i.e., language/dialect-specific phonetics), there are also articulatory patterns at the individual level that are likely independent of vocal tract anatomy and physiology.

In monolingual talkers, these group-level and individual-level patterns of speech production are inextricably linked; however, in bilingual talkers, these two sources of variability—language/dialect-specificity and talker-specificity—are potentially decoupled. This decoupling then raises the question of how language/dialect-specific and talker-specific speech production patterns interact in bilingual talkers. In particular, we ask whether there are any language-independent, talker-specific characteristics that are consistently evident in each of the two languages of a bilingual talker, and that, in combination with the anatomically or physiologically determined features, become part of the unique set of indexical features for that individual regardless of the language being spoken. Such features could be either acquired (i.e., experience-related and learned) or a consequence of more central, rather than peripheral, control processes in language and speech production. For example, working memory, IQ, and, lexical retrieval speed, amongst other cognitive and/or linguistic characteristics, may influence both first-language (L1) and second-language (L2) speech production. It is also possible that individual personality traits may bear some relation to patterns of speech production (e.g., see Dewaele and Furnham, 1999, 2000, for a review of studies indicating greater fluency in the speech of extroverts relative to the speech of introverts in both L1 and L2). These individual traits would thus contribute a consistent talker-specific influence on both first- and second-language speech production.

Three lines of prior research bear on the relationship between language- and talker-specificity in speech production by bilingual talkers. First, there is a wealth of research demonstrating L1 and L2 interactions at various levels of speech production and perception (for a review, see Davidson, 2011). These cross-language interactions manifest throughout the phonetic system at the segmental and supra-segmental levels, including vowel systems (e.g., Guion, 2003; Chang, 2012, 2013; Mayr *et al.*, 2012), *F*0 level (e.g., Yoon, 2015), and *F*0 alignment (e.g., Mennen, 2004). The present study aims to complement these data and models by looking for L1-L2 interactions in the long-term acoustic features (i.e., utterance—rather than sublexical—or lexical levels) that do not directly convey linguistically meaningful, contrastive information, but that may instead convey indexical information for language, group, and talker identification. That is, in this study we ask whether, in addition to the well-documented mutual influence of the L1 and L2 sound systems on speech sound production, there is a dependency between global acoustic features of L1 and L2 speech within individual talkers regardless of the particular L1 and L2 in question.

A second major advance in phonetic theory that underlies the present study is the steady accumulation of evidence that all acoustic-phonetic dimensions of the speech signal simultaneously convey information about what is being said (linguistic information about the utterance), as well as about who is saying it (indexical information about the talker). For example, using sine-wave replicas of English speech in which the spectro-temporal dynamics of the signal (i.e., acoustic properties traditionally considered phonetic cues) are preserved but the vocal source characteristics (i.e., acoustic properties traditionally considered talker cues) are removed, Remez *et al.* (1997) showed that listeners can identify talkers based on phonetic cues alone. Moreover, other research has shown that listeners are highly sensitive to talker-specificity in segment-level production (e.g., Allen and Miller, 2004), and that speech-in-noise is better recognized when spoken by familiar rather than unfamiliar talkers following talker identification training (e.g., see Nygaard *et al.*, 1994, for an early and powerful demonstration of speech perception as a talker-contingent process). Taken together, studies such as these provide evidence against a model of speech processing and representation in which recognition of linguistic and indexical information rely on separate acoustic features with each process relying on the perceptual separation and discarding of acoustic properties that are not relevant for the current task (i.e., either word or talker identification).

The integration of linguistic (contrastive phonetic) and indexical (talker) information suggests that some degree of idiolectal variability is likely constrained by language-specific structural features (e.g., phoneme inventory size and structure, phonotactic patterns, etc.). In the case of bilingual talkers, for whom two linguistic sound systems must coexist, this may mean that the need to maintain a greater number of phonetic distinctions along any given acoustic dimension might further constrain the range of idiolectal variability. (Note that this account assumes integrated L1-L2 phonetic systems with constant co-activation of both languages.) Moreover, acoustic-phonetic variation along global acoustic-phonetic dimensions that are shared across a bilingual's L1 and L2 may be independently controlled in the two languages. For example, within a given language, speaking rate (in terms of syllables per second) may be substantially constrained by dialectal affiliation (e.g., see Jacewicz *et al.*, 2010, for evidence of dialect-based variation in American English), and therefore an individual bilingual talker may have a relatively fast speaking rate in one language but a relatively slow speaking rate in the other language depending on the characteristic temporal patterns of the talker's dialectal affiliation in each language. Consistent with this view, Wilson and Gick (2013) demonstrated that highly proficient, balanced bilinguals adopted distinct language-specific articulatory settings for each language. This study demonstrated distinct interspeech postural settings (i.e., lip, jaw, and tongue position during inter-utterance pauses) in French and English sentence recordings by a group of French-English bilinguals, indicating that bilinguals can switch between the language-specific articulatory settings of their two languages. However, this finding does not preclude the possibility that there are other idiolectal, global speaking style characteristics that are not entirely independently controlled in the two languages of bilinguals such that some aspects of L1 speech are significant predictors of L2 speech within individual bilingual talkers. The present study investigates this possibility of language-independent talker-specificity in bilinguals.

A third line of research that bears on the issue of language- and talker-specificity in bilingual speech production addresses cross-language talker identification. Several studies have investigated talker identification across listeners with varying degrees of familiarity with the language being spoken (Thompson, 1987; Goggin *et al.*, 1991; Perrachione and Wong, 2007; Winters *et al.*, 2008). These studies have shown that talker identification in a known language is more accurate than talker identification in an unknown language. This language-familiarity advantage for talker identification suggests that the manifestation of talker-specificity in speech production is constrained by the language-specific sound system. This claim is further supported by evidence that better language-specific phonological processing abilities confer a talker identification benefit for individual listeners (Perrachione *et al.*, 2011). This study showed that listeners with impaired phonological processing (listeners with dyslexia) were impaired relative to control listeners on a voice identification task in their native language. Nevertheless, it is important to note that language-independent talker identification accuracy was reported to be significantly above chance for bilingual talkers (Winters *et al.*, 2008), and both listeners with and without dyslexia were moderately successful at a voice recognition task with speech in an unfamiliar language (Perrachione *et al.*, 2011). These data could be accounted for by assuming that better-than-chance talker identification accuracy, regardless of the listener's familiarity with the language of the speech sample, can be accomplished purely on the basis of cues that are related to the talker's vocal tract anatomy and physiology. In this account, the language-familiarity advantage (and similarly, the impaired phonological processing disadvantage for listeners with dyslexia) reflects the benefit of knowledge on the part of the listener of the possible range of idiolectal variations within the constraints of the sound system of the particular language. However, for bilingual talkers, there may be additional talker-specific idiolectal characteristics that are independent of language-specific phonetic and phonological constraints, and that reflect a talker-specific speaking characteristic that comes into play for both L1 and L2 production. These talker-specific, language-independent phonetic consistencies, which may be more centrally controlled and therefore independent of the features determined by the talker's vocal tract, could then contribute to the set of cues that facilitates bilingual talker identification across the two languages of a bilingual talker.

The present study seeks evidence for language-independent, talker-specific characteristics by examining a global, talker-controlled feature across both languages of a group of bilingual talkers. Specifically, we examined speech-timing patterns in a corpus that includes comparable recordings in each of the two languages of a group (*n* = 86) of linguistically diverse bilinguals (L2 = English, L1 = one of ten different languages). We chose to focus primarily on timing in terms of speaking rate (operationalized as number of intensity peaks, or “acoustic syllables” per second) because it can be automatically and consistently measured across languages. Moreover, as a global feature that sets a temporal frame (or tempo) for an utterance rather than conveys phonemic contrasts, speaking rate is exactly the type of acoustic feature that could be subject to both language/dialect- and talker-specific control. It is also more likely to be independent of the anatomical and physiological constraints of a particular vocal tract than other global features such as fundamental frequency or the long-term average speech spectrum. This set of conditions thus allows for the possibility of a dissociation between average L1 and L2 speaking rates in absolute terms (with L2 speaking rate invariably being slower than L1 speaking rate), but an association of relative L1 and L2 speaking rates across a group of bilingual talkers (i.e., relatively fast L1 talkers may also be relatively fast L2 talkers). The dissociation of average rates in L1 and L2 would establish that the parameter in question (speaking rate) is not an automatic consequence of the talker's vocal anatomy and physiology, but is instead a learned and/or centrally (rather than peripherally) controlled property. This then provides the necessary condition for investigating the relationship between relative L1 and L2 speaking rates as an indicator of the relationship between language- and talker-specificity in bilinguals.

There is some previous evidence that L2 measures of oral fluency, such as number of filled pauses, number and duration of silent pauses, and speaking rate, are related to L1 fluency (Towell and Dewaele, 2005; Derwing *et al.*, 2009; De Jong *et al.*, 2015) and to the general notion of an individual speaking style (e.g., Kormos, 1999, on individual speech monitoring style). Motivated by the need to develop valid, accurate, and ultimately automatic measures of L2 fluency, this prior work contributed important evidence that some of the variation in L2 fluency can be accounted for by variation in L1 fluency, and has led to the recommendation that L2 acquisition research and the assessment of L2 proficiency should involve measurement of both L1 and L2 fluency so that L2-specific performance can be accounted for independently of talker-specific speech and language traits (Segalowitz, 2010). Specifically, Derwing *et al.* (2009) reported a significant correlation between L1 and L2 fluency (based on subjective listener ratings), as well as between L1 and L2 temporal measures (in terms of number of pauses and speech rate) for 16 Mandarin and 16 Slavic (Russian and Ukranian) learners of English. However, these L1-L2 correlations weakened across time in this longitudinal study and were overall stronger for the Slavic than for the Mandarin learners of English. Similarly, in a study with 29 English-speaking and 24 Turkish-speaking learners of Dutch, De Jong *et al.* (2015) reported that L2 fluency could be predicted on the basis of L1 fluency (where fluency was assessed in terms of various measures, including mean syllable duration, pause characteristics, including number and duration of pauses within and between speech units, number of filled and silent pauses, number of repetitions and corrections). And Towel and Dewaele (2005) found a strong positive correlation between L1 and L2 speaking rates (syllables/min) in a group of 12 L2 learners (L1 English, L2 French). A primary concern of these previous studies was in teasing apart the influence of speaking style (a talker-inherent trait) from the influence of proficiency (a dynamic property related to the process of L2 acquisition) on L2 fluency. In the current study we arrived at the question of an L1-L2 correlation through the lens of work on the integration of, and listener sensitivity to, indexical and linguistic information in the speech signal. As such, we focus exclusively on speech rate, which can be automatically extracted from speech recordings in any language without prior transcription and text-to-signal alignment. We also include bilingual English talkers from ten different L1 backgrounds as a means of increasing the generalizability of the findings and minimizing the influence of the sound structures of particular languages (i.e., minimizing specifics of L1 to L2 transfer that may hide or distort talker- and/or L1/L2 status-specificity).

The strategy that we adopt in our analyses involves three steps. First, we compared speaking rate across the various languages represented in our corpus to determine the extent of variability across L1 talkers of various languages. Though not a primary concern for the present study, which focuses on the relationship between L1 and L2 speech production in bilinguals, this first step in the analysis provides an indication of cross-language variation in average speaking rate (see Pellegrino *et al.*, 2011, for extensive discussion of rate variation across languages) and provides an essential point of comparison for the subsequent measures of L2 speaking rate. Second, we compared speaking rates across L1 and L2 speech. We examined this L1-L2 difference in two distinct analyses, one compares L1 and L2 English (monolinguals vs bilinguals) while the other compares L1 and L2 speech within bilinguals (various L1s vs L2 English). These L1-vs-L2 analyses, within English (across individuals) and within bilinguals (across their two languages), were expected to replicate the well-established slower speaking rate of L2 speech compared to L1 speech (e.g., Guion *et al.*, 2000; Baese-Berk and Morrill, 2015). Finally, in the critical analysis for the present study, we investigated whether L1 speaking rate is a significant predictor of L2 speaking rate within the group of bilingual talkers. A positive finding would suggest that the general slowing associated with L2 speech production occurs in proportion to an individual's L1 speaking rate, and therefore that talker-specific characteristics in L1 speech prevail in L2 speech. Put another way, this finding would indicate that some significant portion of individual L2 variation can be traced to individual variation in L1, a source of individual variation that is often overlooked in studies of L2 speech production and perception [though, as noted above, a relationship between L2 fluency and L1 fluency has received some attention in the literature on Second Language Acquisition (SLA) for the purposes of valid and accurate L2 proficiency assessment, e.g., Towel and Dewaele, 2005; Derwing *et al.*, 2009; De Jong *et al.*, 2015].

In the present study, we investigate overall speaking rate across bilingual English speakers from various L1 backgrounds and under various task demands. The inclusion of various speech recording tasks, including a reading passage, a picture narrative task, and a free-form question-and-answer speech elicitation task, allows us to view L2 related speaking rate adjustments under task demands that vary in their emphasis on speech articulation vs more complex language production. A difference in the strength of L1-L2 speaking rate association across tasks would suggest that language-independent talker-specific differences are constrained by task and linguistic demands. In particular, we hypothesized that language-independent talker-specificity in a global phonetic property such as overall speaking rate in bilinguals may be more likely to emerge in tasks that involve complex language generation (free-form question-and-answer and story narratives) than in a task that emphasizes speech production without language generation at the conceptual level (paragraph reading).

## II. METHOD

### A. Materials

Recordings for this study were all taken from a corpus of digital speech recordings that includes both read speech and spontaneous speech by bilingual and monolingual English speakers. A key feature of this corpus is that it includes recordings in both the L1 and the L2 (English) of each bilingual talker. All talkers in the corpus were recorded producing a common set of materials that was selected to cover both read and spontaneous speech, and to be comparable across languages. The full set of recordings in each language consisted of six distinct subsets, three of which were sentence lists, one of which was a paragraph-length reading passage, and two of which were designed to elicit spontaneous speech. The sentence recordings are intended primarily for use as stimuli in speech perception experiments and were not analyzed for the present study. The paragraph is the widely translated fable, “The North Wind and the Sun,” as available for all languages included in the *Handbook of the International Phonetic Association* (1999). The spontaneous speech recordings were recorded in response to two types of prompts. The first involved a series of published picture stories (Mayer, 1973, 1974a,b) that can be verbalized into an oral story for recording. These cartoons are culturally neutral and involve animals who find themselves in humorous or otherwise charming situations. No translation is required prior to recording since these story prompts are purely visual. Two such cartoons were designated for the L1 recordings: “Bird's New Hat” (Mayer, 1974a), and “Bubble Bubble” (Mayer, 1973). Another two were designated for the L2 recordings: “Just a Pig at Heart” (Mayer, 1974b) and “Bear's New Clothes” (Mayer, 1974a). For the purpose of acoustic analysis, the two picture narratives for each language by each talker were digitally concatenated into one recording. The second spontaneous speech prompt involved a list of questions that were intended to elicit a monologue of approximately five minutes. These questions were composed in English and then translated into the other languages by personal acquaintances of the experimenters. For each language, one person translated the sentences from English into their native language, and a second person provided a back translation into English. The resulting English translations were compared to the original English for authenticity. Adjustments were made as necessary and as agreed upon by the two native speakers. The questions probe common topics of conversation between acquaintances, including information about the talker's family and place of origin, holiday celebrations, impressions of their current location, and food and recreational preferences.

To date, this corpus (the ALLSSTAR corpus, or Archive of L1 and L2 Scripted and Spontaneous Transcripts and Recordings) includes recordings from 119 bilingual talkers from 21 different L1 backgrounds, plus 27 monolingual English talkers who provided English recordings only. Additional and current information about the continuously updated and expanded corpus can be found online.^1^ Access to the recordings and the speech elicitation materials is available upon request via the Online Speech/Corpora Archive and Analysis Resource at Northwestern University (OSCAAR^2^). Orthographic transcriptions of the spontaneous speech recordings are stored alongside the digital speech files in OSCAAR (wherever available).

In the present study, we analyzed the recordings of the paragraph-length reading passage (NWS), the picture story narratives (ST), and the open response questions (Q&A) by 86 bilinguals in both of their languages and by 27 monolingual English talkers (English only).

### B. Talkers and recording procedure

The bilingual talkers included in this study came from ten different L1 backgrounds (listed in Table I). L1s included in the ALLSSTAR corpus for which we had fewer than four talkers were excluded from the present analysis. We also analyzed the matched English recordings from the English monolinguals (*n* = 27) in the corpus.

**TABLE I.** Talkers from the ALLSSTAR corpus included in the present study.

	*F*	*M*	Total
Cantonese	8	6	14
English (monolinguals)	14	13	27
Hebrew	1	3	4
Hindi	1	4	5
Korean	7	4	11
Mandarin	3	11	14
Portuguese (Brazilian)	3	2	5
Russian	1	4	5
Spanish	3	8	11
Turkish	2	11	13
Vietnamese	3	1	4
Total (bilinguals + monolinguals)			113
Total (bilinguals)			86

All bilingual and monolingual talkers were recruited from the graduate student population at Northwestern University. All were paid for their participation or received course credit. All reported normal speech and hearing at the time of testing and were 18–34 years of age (average of 23 yr). While English proficiency of the bilingual talkers varied, all talkers had achieved a level of English language proficiency that was sufficient to gain entry into a graduate program at Northwestern. Nevertheless, most of these bilingual talkers were enrolled, either by choice or by requirement, in intensive English language instruction as offered by the Northwestern University English Language Programs. Standardized English test scores (TOEFL, SPEAK, and/or Versant English Test^3^) were available for many, though not all, of the bilingual participants through self-report or by consented release from the Northwestern University English Language Programs.

Participants were recorded in a sound-treated booth. They spoke into a Shure SM81 Condenser microphone (Shure Inc., Niles, IL) and their speech was recorded direct to disk onto an Intel Core 2 Duo iMac (Intel, Santa Clara, CA). All talkers completed a language background questionnaire before beginning the recording of the sentences, paragraph, and spontaneous speech in English. The bilingual talkers returned the following day for a second recording session during which they recorded the sentences, paragraph, and spontaneous speech recordings in their native language (the L1 recordings). All scripted materials were presented in the standard orthography of the language. Each session took ∼1–1.5 h.

### C. Acoustic measurements

The primary measure of interest for the current study was speaking rate, which we measured in both the L1 and L2 speech samples from the read speech (NWS paragraph) and spontaneous speech recordings (picture story narratives, ST, and Q&A). We obtained two measures of speaking rate, speech rate and articulation rate, from each of the three speech samples in each language, both of which were based directly on the number of (acoustic) syllables (i.e., intensity peaks) per second. The two speaking rate measures differed only with respect to the inclusion (speech rate) or exclusion (articulation rate) of silent pauses.

From each speech sample we obtained the number of syllables using an automatic syllable detection algorithm implemented as a Praat script (De Jong and Wempe, 2009). This script counts the number of intensity peaks in a digitized speech signal that are preceded and followed by intensity troughs, excluding peaks that are not voiced. For the first measure of speaking rate, the total number of peaks (syllabic nuclei) was divided by the duration of the recording with major disfluencies (e.g., coughs) excluded. We refer to this measure as “speech rate.” For the second measure of speaking rate the total number of peaks (syllabic nuclei) was divided by the duration of the recording with major disfluencies (e.g., coughs) and silent pauses of at least 100 ms in duration excluded. We refer to this measure as “articulation rate.”

Additionally, we obtained the average number of syllables per utterance where utterance was defined as a stretch of speech surrounded by pauses of at least 100 milliseconds. This measure of average utterance length (i.e., the average number of syllables produced from one pause to the next, or number of syllables/pause) was included as a predictor of speech rate and articulation rate in the statistical analyses because prior work (e.g., Quené, 2008; Jacewicz *et al.*, 2010) indicated a strong positive relationship between speaking rate and utterance length.^4^

### D. Statistical analyses

The speech rate and articulation rate data were analyzed with linear mixed effects regression models using the lme4 (Bates *et al.*, 2015) and the languageR packages (Baayen, 2013). Five hypotheses were each tested with a multivariate regression model with two dependent measures, speech rate (pauses included) and articulation rate (pauses excluded).^5^ In order to ensure a fair comparison between the two measures of speaking rate (speech rate and articulation rate), measurements for all analyses were *z*-transformed within their own distributions [i.e., within each measure (speech rate or articulation rate) and within the data for each task (NWS, ST, Q&A)]. The specific dependent variable entered into each statistical model was a set of such *z*-transformed acoustic measurements. It is important to note that, while an untransformed speech rate measure is necessarily always slower than the matched untransformed articulation rate measure (they have the same numerator but different denominators), a *z*-transformed speech rate measure can be either larger, equal to, or smaller in magnitude than its *z*-transformed articulation rate counterpart due to the fact that the duration of pauses varies substantially within stretches of speech. Therefore, a given talker's average articulation rate measure (pauses excluded) may be close to the group-wise average articulation rate measure, but this same talker's average speech rate measure (pauses included) may be quite far (in either direction) from the group-wise average speech rate measure. This would indicate that the talker in question exhibits quite typical articulation rate but rather atypical pausing behavior relative to the group.

For all analyses, the fit of the base model (i.e., the model that includes only the predictors in the hypothesis being tested) was compared to the fit of additional models that included additional predictors (i.e., predictors that are not part of the explicit hypothesis being tested, e.g., age, gender, utterance length, task, and language status). Models were compared by means of the anova function from the lmerTest package (Kuznetsova *et al.*, 2015). Note that inclusion of a predictor may significantly improve the overall model fit (relative to the base model) even though its separate influence on the dependent variable may not rise to a significant level in the analysis result. Below, we report results of the models with the largest log likelihood. The models also included the maximal random effects structure supported by the data. The multicollinearity of the models was measured with the kappa.mer function (Frank, 2014).

The specific hypotheses we tested are as follows:

H1. L1 speaking rate varies across languages
H2a. L1 English speaking rate by monolinguals is faster than L2 English speaking rate by bilinguals
H2b. Within bilingual talkers, L1 speaking rate is faster than L2 speaking rate
H3a. Within bilingual talkers, L1 speaking rate predicts L2 speaking rate
H3b. Within bilingual talkers, L1 speaking rate predicts L2 speaking rate, when controlled for proficiency levels

The overall structure of the data set for these analyses consisted of multiple entries for each talker in each language (only one language for the monolinguals) with each entry representing a speaking rate measure (either speech rate or articulation rate, *z*-transformed as described above) for one of the three tasks (NWS, ST, Q&A).

First, we asked whether L1 speaking rate varied across languages (H1, L1-L1 comparison). For this analysis, the fixed effect factors in the best fitting, maximal model were L1 (all 11 languages listed in Table I), task (NWS, ST, Q&A), measure (speech rate, articulation rate), talker age, and utterance length. All possible two- and three-way interactions among L1, task, and measure were also included as fixed effect factors. L1 was effects coded, with English as the baseline and all other L1s compared to the grand mean. Task and measure were contrast coded, with task coded in two ways, NWS vs STQ&A for a read vs spontaneous speech comparison, and ST vs Q&A for a comparison of directed vs more free-form spontaneous speech. Age and utterance length were centered. The random intercept was talker, with measure and utterance length as random slopes. The multicollinearity of the model was moderate with a condition number of 14.87.

Second, we asked whether speaking rate differed across L1 speech and L2 speech. To address this question, we conducted two separate analyses: one within English (H2a, L1-English by the monolinguals vs L2-English by the bilinguals), and a second within bilingual talkers (H2b, various L1s vs L2-English). In the first regression model (L1-English vs L2-English across monolingual and bilingual talker groups), the fixed effect factors of the best fitting, maximal model were language status (L1, L2), task (NWS, ST, Q&A), measure (speech rate, articulation rate), and utterance length. All possible two- and three-way interactions among language status, task, and measure were also included as fixed effect factors. Language status, task, and measure were contrast coded; utterance length was centered. The random intercept was talker, with task and measure as random slopes. The multicollinearity of this model was low, with a condition number of 3.77. In the second regression model (various L1s vs L2-English within bilinguals), the fixed effect factors of the best fitting, maximal model were language status (L1, L2), task (NWS, ST, Q&A), measure (speech rate, articulation rate), L1 (the 10 language groups listed for the bilinguals in Table I), utterance length, and talker gender. All possible two-, three-, and four-way interactions among L1, measure, language status, and task were also included as fixed effect factors. Language status, task, measure, and gender were contrast coded; utterance length was centered. L1 was effects coded with Cantonese as the baseline and all other L1s compared to the grand mean. The random intercept was talker, with utterance length and measure as random slopes for talker. The multicollinearity of this model was low, with a condition number of 9.17. For both of these models, task was coded in two ways: NWS vs STQ&A (read vs spontaneous speech), and ST vs Q&A (within spontaneous speech, directed vs more free-form).

Finally, we asked whether individual bilingual talkers' L2 speaking rates could be predicted by their L1 speaking rates (H3a). In this multivariate analysis, the dependent variables were L2 speech rate and L2 articulation rate. The dataset consisted of multiple entries for each talker in each language with each entry representing a speaking rate measure (either speech rate or articulation rate, *z*-transformed as described above in the first paragraph of Sec. II D) for one of the three tasks (NWS, ST, Q&A). Two bilingual talkers' data (one Chinese and one Russian) were excluded from this analysis because they lacked either L1 speaking rate or L2 speaking rate for one of the tasks. Therefore, 84 bilingual talkers' data were used in this analysis. The best fitting model's fixed effect factors were L1 rate, task (NWS, Q&A, ST), measure (speech rate, articulation rate), L1 utterance length, L2 utterance length, talker gender, and L1 (the ten language groups listed for the bilinguals in Table I). Additionally, a set of all possible two- and three-way interactions among L1 rate, measure, and task and another set of all possible two- and three-way interactions among L1, measure, and task were included as fixed effect factors. L1 was effects coded with Cantonese set as the baseline and all other L1s compared to the grand mean. Task, measure, and gender were contrast coded and the remaining measures were centered. The random intercept was talker, with task included as the random slope. The multicollinearity of this model was low, with a condition number of 9.95.

A separate model was run with inclusion of Versant test scores (where available) to test for the influence of L2 (English) proficiency on L2 rate, and to see if the effect of L1 rate on L2 rate remains when variation in L2 proficiency is controlled (H3b). In this model, the fixed effect factors were L1 rate, task (NWS, Q&A, ST), measure (speech rate, articulation rate), L1 (the ten language groups listed for the bilinguals in Table I), L1 utterance length, L2 utterance length, and Versant test scores. Three sets of all possible two- and three-way interactions were also included as fixed effect factors: the one among L1 rate, task, and measure, the one among L1, task, and measure, and the one among Versant scores, task, and measure. As for the other models, task was contrast coded in two ways: NWS vs STQ&A (read vs spontaneous speech), and ST vs Q&A (within spontaneous speech, directed vs more free-form). Measure was also contrast coded. L1 utterance length, L2 utterance length, and Versant test scores were centered. L1 was effects coded with Mandarin as the baseline and the other languages compared to the grand mean. (Note that, unlike all other analyses, Cantonese was not set as the baseline in this analysis because Versant test scores were not available for any of the Cantonese-English bilingual talkers.) The random intercept was talker, and task, L2 utterance length, and measure were random slopes. The multicollinearity of this model was moderate with a condition number of 13.74.

## III. RESULTS

Table II shows the average and standard deviations of L1 and L2 speech rates and articulation rates for the read and spontaneous speech samples across all talkers in each of the language groups. (Data for the monolingual English talkers are shown in the top section of Table II only.)

**TABLE II.** Average and standard deviations of L1 (top) and L2 (bottom) speech rates and articulation rates for the reading passage (NWS) and both samples of spontaneous speech (ST and Q&A) across all talkers in each of the language groups.

	Speech rate (syllable/second with silence included)						Articulation rate (syllable/second with silence excluded)					
	NWS		Q&A		ST		NWS		Q&A		ST	
	Average	Standard deviation	Average	Standard deviation	Average	Standard deviation	Average	Standard deviation	Average	Standard deviation	Average	Standard deviation
Cantonese	3.44	1.11	3.07	1.29	3.23	0.89	4.65	0.48	4.35	0.48	4.61	0.35
Hebrew	4.14	1.08	2.92	0.49	3.14	0.53	5.25	0.47	4.21	0.32	4.78	0.42
Hindi	3.93	0.95	3.43	0.96	3.43	1.06	4.99	0.39	4.47	0.43	4.64	0.53
Korean	3.94	0.63	3.10	0.99	3.29	0.95	4.92	0.37	4.57	0.30	4.96	0.33
Mandarin	3.77	1.09	3.43	0.83	3.43	1.01	5.11	0.32	4.65	0.42	4.84	0.40
Portuguese (Brazilian)	3.71	1.68	3.42	0.56	3.28	0.75	4.69	0.42	4.62	0.16	4.77	0.21
Russian	3.61	1.08	2.86	0.66	2.63	0.69	5.03	0.32	4.67	0.55	4.75	0.42
Spanish	3.95	1.18	3.24	0.53	3.39	0.75	5.19	0.47	4.69	0.28	4.98	0.37
Turkish	4.02	0.87	3.46	0.84	3.50	0.91	5.24	0.29	4.87	0.20	5.10	0.32
Vietnamese	3.72	0.95	2.88	0.75	2.99	0.71	4.74	0.28	4.26	0.20	4.48	0.51
English	3.83	0.85	3.35	0.92	3.25	1.00	4.82	0.29	4.52	0.37	4.66	0.39
**Average (L1)**	**3.82**	**1.01**	**3.26**	**0.94**	**3.28**	**0.95**	**4.95**	**0.42**	**4.57**	**0.39**	**4.77**	**0.41**
Cantonese	2.80	0.89	2.24	0.93	2.09	0.92	3.89	0.41	3.59	0.51	3.69	0.52
Hebrew	3.40	0.79	2.41	0.56	2.62	0.49	4.45	0.39	3.87	0.36	4.44	0.33
Hindi	3.47	0.98	3.51	1.10	3.12	0.92	4.53	0.22	4.39	0.24	4.45	0.36
Korean	2.97	0.89	2.12	0.93	1.95	0.78	3.97	0.39	3.66	0.47	3.76	0.41
Mandarin	3.18	1.00	2.79	0.88	2.64	0.83	4.26	0.36	3.93	0.50	4.11	0.40
Portuguese (Brazilian)	2.95	1.15	2.61	0.53	2.33	0.79	4.05	0.17	3.74	0.34	3.89	0.26
Russian	3.27	0.52	1.92	0.79	1.97	0.35	4.71	0.22	3.54	0.83	4.19	0.23
Spanish	3.04	1.07	2.50	1.13	2.48	0.72	4.23	0.29	4.08	0.44	4.19	0.34
Turkish	3.12	1.03	2.66	0.56	2.42	0.81	4.21	0.28	4.04	0.23	4.17	0.44
Vietnamese	3.11	0.86	2.64	0.54	2.29	0.62	4.01	0.14	3.92	0.36	3.91	0.28
**Average (L2)**	**3.08**	**0.88**	**2.52**	**1.03**	**2.37**	**0.87**	**4.18**	**0.39**	**3.87**	**0.50**	**4.04**	**0.46**

The data in this table are presented in terms of the number of syllables per second; however, as described above in the first paragraph of Sec. II D, for the statistical analyses individual syllable rates were *z*-transformed so that a fair comparison between speech rate (includes silent pauses) and articulation rate (excludes silent pauses) could be performed. The analysis of speaking rate differences across the various L1s showed that all of the main effects (L1, task, measure, and utterance length) reached significance except for talker age. (See Appendix A, Table III, for the estimate, standard error of the estimate, and significance level for all significant fixed effects in this model.) The main effect of L1 was due to the faster rate of Turkish compared to the grand mean across all 11 languages. The main effect of task was due to faster rates for read than for spontaneous speech, and within spontaneous speech, faster rates for ST than Q&A. The articulation rate measure (*z*-transformed) was significantly faster than the speech rate measure (*z*-transformed). Finally, as expected based on prior work (e.g., Quené, 2008; Jacewicz *et al.*, 2010), utterance length positively predicted rate; that is, longer utterances had faster rates than shorter utterances. There was a significant interaction between L1 and measure, with seven languages (Cantonese, Mandarin, Hebrew, Korean, Brazilian Portuguese, Spanish, and Turkish) showing a slower speech rate than articulation rate, whereas this difference was reversed for Russian, and non-significant for the remaining languages. This cross-language variation in the difference between *z*-transformed articulation rate and *z*-transformed speech rate indicates variation in pausing behavior, which could be due to individual talker and/or structural linguistic variation. A more fine-grained analysis of the nature of this variation in pausing behavior (e.g., variation in the number, duration, and preferred syntactic location of pauses) is beyond the scope of the present study as it would require a detailed analysis of the morpho-phonological and syntactic features of the various languages. There was also a significant L1 by task interaction, involving either a relatively small read-spontaneous difference (Russian), a relatively great read-spontaneous difference (Korean and Turkish), or a different-from-typical difference within the two types of spontaneous speech (Russian and Hebrew). The task by measure interaction was also significant, as was the three-way interaction between L1, task, and measure, due to the fact that two languages (Mandarin and Hindi) showed a smaller read-spontaneous difference in speech rate than in articulation rate, whereas two other languages (Korean and Russian) showed a greater read-spontaneous difference in speech rate than in articulation rate. Overall, while this analysis across L1s showed some notable patterns, there was no clear, interpretable, and systematic trend that could be taken as a reliable indicator of a strong language-specific effect on speaking rate (however, see Pellegrino *et al.*, 2011, for more discussion of cross-language rate variation). For the present purpose, these L1 data serve as points of comparison for the subsequent analyses.

**TABLE III.** Significant fixed effects for the analysis of speaking rate across various L1s (see Table I for the languages included). Significance codes: *** = 0; ** = 0.001; * = 0.01. Dependent variables were speech rate and articulation rate, *z*-transformed within their own distributions. Random intercept was talker, with measure and utterance length as random slopes.

	Estimate	Standard Error	df	*t*-value	Pr (>|*t*|)	
(Intercept)	−0.05	0.06	107	−0.78	0.44	
Measure	−0.46	0.06	107	−7.18	0.00	***
Task (NWS vs Q&A + ST)	−0.59	0.10	124	−5.68	0.00	***
Task (Q&A vs ST)	0.24	0.04	106	5.85	0.00	***
L1 Turkish	0.44	0.13	100	3.36	0.00	**
Utterance length	0.16	0.01	623	13.86	<2 × 10^−16^	***
Measure:Task (NWS vs Q&A + ST)	0.46	0.09	530	5.25	0.00	***
Measure:Task (Q&A vs ST)	0.39	0.07	522	5.66	0.00	***
Measure:L1 Cantonese	0.48	0.15	106	3.19	0.00	**
Measure:L1 Mandarin	0.70	0.15	106	4.68	0.00	***
Measure:L1 Hebrew	0.67	0.26	106	2.55	0.01	*
Measure:L1 Korean	0.74	0.17	106	4.45	0.00	***
Measure:L1 Portuguese	0.47	0.24	106	2.01	0.05	*
Measure:L1 Russian	−6.01	0.24	115	−24.68	<2 × 10^−16^	***
Measure:L1 Spanish	0.87	0.17	106	5.25	0.00	***
Measure:L1 Turkish	0.92	0.15	106	5.96	0.00	***
Task (Q&A vs ST):L1 Hebrew	0.43	0.17	106	2.61	0.01	*
Task (NWS vs Q&A + ST):L1 Korean	0.87	0.25	102	3.40	0.00	***
Task (NWS vs Q&A + ST):L1 Russian	−2.88	0.37	109	−7.69	0.00	***
Task (Q&A vs ST):L1 Russian	−0.84	0.16	107	−5.09	0.00	***
Task (NWS vs Q&A + ST):L1 Turkish	0.52	0.23	99	2.19	0.03	*
Measure:Task (NWS vs Q&A + ST):L1 Mandarin	−0.75	0.20	524	−3.68	0.00	***
Measure:Task (NWS vs Q&A + ST):L1 Hindi	−0.67	0.32	523	−2.08	0.04	*
Measure:Task (NWS vs Q&A + ST):L1 Korean	0.45	0.23	523	2.00	0.05	*
Measure:Task (NWS vs Q&A + ST):L1 Russian	1.93	0.34	568	5.60	0.00	***

For the comparisons of speaking rate across L1 and L2 speech, we first compared L1 English by monolingual talkers with L2 English by bilingual talkers. All of the main effects in this analysis reached significance: language status, task (read faster than spontaneous; and within spontaneous, ST faster than Q&A), measure (articulation rate faster than speech rate), and utterance length (faster rates for longer utterances). (See Appendix B, Table IV, for the estimate, standard error of the estimate, and significance level for all significant fixed effects in this model.) There was a significant two-way interaction between language status and task such that the L1-L2 speaking rate difference was smaller in spontaneous speech (Q&A and ST) than in read speech (NWS). Moreover, there was a significant two-way interaction between measure and task, with slightly greater task-dependent differences for speech rate than for articulation rate. None of the other interactions were significant. Most importantly for the current study, L1 English speech by monolinguals had a consistent and reliably faster rate than L2 English speech by bilinguals (main effect of language status).

**TABLE IV.** Significant fixed effects for the comparisons of speaking rate of L1 English by monolingual talkers with L2 English by bilingual talkers. Significance codes: *** = 0; ** = 0.001; * = 0.01; . = 0.05. Dependent variables were speech rate and articulation rate, *z*-transformed within their own distributions. Random intercept was talker, with task and measure as random slopes.

	Estimate	Standard Error	df	*t*-value	Pr (>|*t*|)	
(Intercept)	0.11	0.06	19	1.92	0.06	.
Measure	−0.16	0.06	19	−2.67	0.01	**
Task (NWS vs Q&A + ST)	−0.22	0.10	46	−2.29	0.02	*
Task (Q&A vs ST)	0.17	0.05	4	3.36	0.00	**
Language status (L1 vs L2)	−0.70	0.12	29	−5.73	0.00	***
Utterance length	0.18	0.01	24	14.67	<2 × 10^−16^	***
Measure:Task (NWS vs Q&A + ST)	0.64	0.08	44	7.50	0.00	***
Measure:Task (Q&A vs ST)	0.50	0.06	46	8.00	0.00	***
Task (NWS vs Q&A + ST): Language status (L1 vs L2)	−0.60	0.17	11	−3.49	0.00	***

Next, we compared L1 and L2 speech within bilingual talkers (data from various L1s shown in the top section of Table I vs matched L2 English in the bottom section of Table I). Significant main effects were found for language status (L1 faster than L2), L1 (Korean had slower rates and Turkish had faster rates compared to the grand mean across all ten languages), task (read faster than spontaneous; and within spontaneous, ST faster than Q&A), and utterance length (faster rates for longer utterances). (See Appendix C, Table V, for the estimate, standard error of the estimate, and significance level for all significant fixed effects in this model.) Measure and gender were also included in this analysis but neither showed a main effect. Language status interacted with L1 (greater L1-L2 differences for Korean and Turkish, and smaller L1-L2 difference for Hindi compared to the other languages), task (smaller L1-L2 difference for spontaneous speech, Q&A and ST, than for read speech, NWS), and measure (smaller L1-L2 difference for speech rate than for articulation rate), and the three-way interaction among language status, L1 and task was also significant (smaller task-related L1-L2 difference variations for Mandarin and Hebrew, and greater task-related L1-L2 difference variations for Korean and Russian). In addition, the three-way interaction among L1, task, and measure was also significant. Finally, there was a significant two-way interaction between measure and task. No other two- or three-way interactions were significant. Most importantly for the current study, L1 speech (various languages) had a consistent and reliably faster rate than L2 English speech in this within-talker analysis.

**TABLE V.** Significant fixed effects for the comparison of speaking rate of L1 (various languages) and L2 English within bilingual talkers. Significance codes: *** = 0; ** = 0.001; * = 0.01. Dependent variables were speech rate and articulation rate, *z*-transformed within their own distributions. Random intercept was talker, with task and measure as random slopes.

	Estimate	Standard Error	df	*t*-value	Pr (>|*t*|)	
(Intercept)	0.01	0.05	76	0.25	0.80	
L1 Korean	−0.25	0.11	71	−2.31	0.02	*
L1 Turkish	0.36	0.10	74	3.57	0.00	***
Task (NWS vs Q&A + ST)	−0.46	0.05	1074	−9.19	<2 × 10^−16^	***
Task (Q&A vs ST)	0.25	0.03	1071	8.19	0.00	***
Language status (L1 vs L2)	−0.66	0.04	1101	−17.97	<2 × 10^−16^	***
Utterance length	0.18	0.01	165	12.89	<2 × 10^−16^	***
L1 Hindi:Measure	−0.36	0.17	80	−2.18	0.03	*
L1 Russian:Measure	0.61	0.17	84	3.58	0.00	***
L1 Korean:Task (NWS vs Q&A + ST)	0.29	0.12	663	2.38	0.02	*
L1 Portuguese:Task (NWS vs Q&A + ST)	0.39	0.14	1050	2.75	0.01	**
L1 Russian:Task (NWS vs Q&A + ST)	−0.74	0.16	762	−4.51	0.00	***
L1 Turkish:Task (NWS vs Q&A + ST)	0.21	0.10	793	2.09	0.04	*
L1 Hebrew:Task (Q&A vs ST)	0.39	0.11	1038	3.51	0.00	***
Measure:Task (NWS vs Q&A + ST)	0.64	0.07	1011	8.58	<2 × 10^−16^	***
Measure:Task (Q&A vs ST)	0.46	0.06	1010	7.79	0.00	***
L1 Hindi:Language status (L1 vs L2)	0.60	0.10	1091	6.18	0.00	***
L1 Korean:Language status (L1 vs L2)	−0.22	0.09	426	−2.40	0.02	*
L1 Turkish:Language status (L1 vs L2)	−0.34	0.08	566	−4.51	0.00	***
Measure:Language status (L1 vs L2)	−0.10	0.05	1013	−2.03	0.04	*
Task (NWS vs Q&A + ST):Language status (L1 vs L2)	−0.18	0.08	1060	−2.28	0.02	*
L1 Mandarin:Measure:Task (NWS vs Q&A + ST)	−0.51	0.16	1010	−3.11	0.00	**
L1 Hindi:Measure:Task (NWS vs Q&A + ST)	−0.61	0.25	1010	−2.39	0.02	*
L1 Korean:Measure:Task (NWS vs Q&A + ST)	0.56	0.18	1010	3.10	0.00	**
L1 Mandarin:Task (NWS vs Q&A + ST):Language status (L1 vs L2)	0.48	0.17	1085	2.80	0.01	**
L1 Hebrew:Task (NWS vs Q&A + ST):Language status (L1 vs L2)	0.65	0.29	1054	2.24	0.02	*
L1 Korean:Task (NWS vs Q&A + ST):Language status (L1 vs L2)	−0.86	0.19	1110	−4.50	0.00	***
L1 Russian:Task (NWS vs Q&A + ST):Language status (L1 vs L2)	−0.78	0.26	1037	−2.96	0.00	**
L1 Russian:Task (Q&A vs ST):Language status (L1 vs L2)	0.68	0.21	1025	3.23	0.00	**

**FIG. 1.**
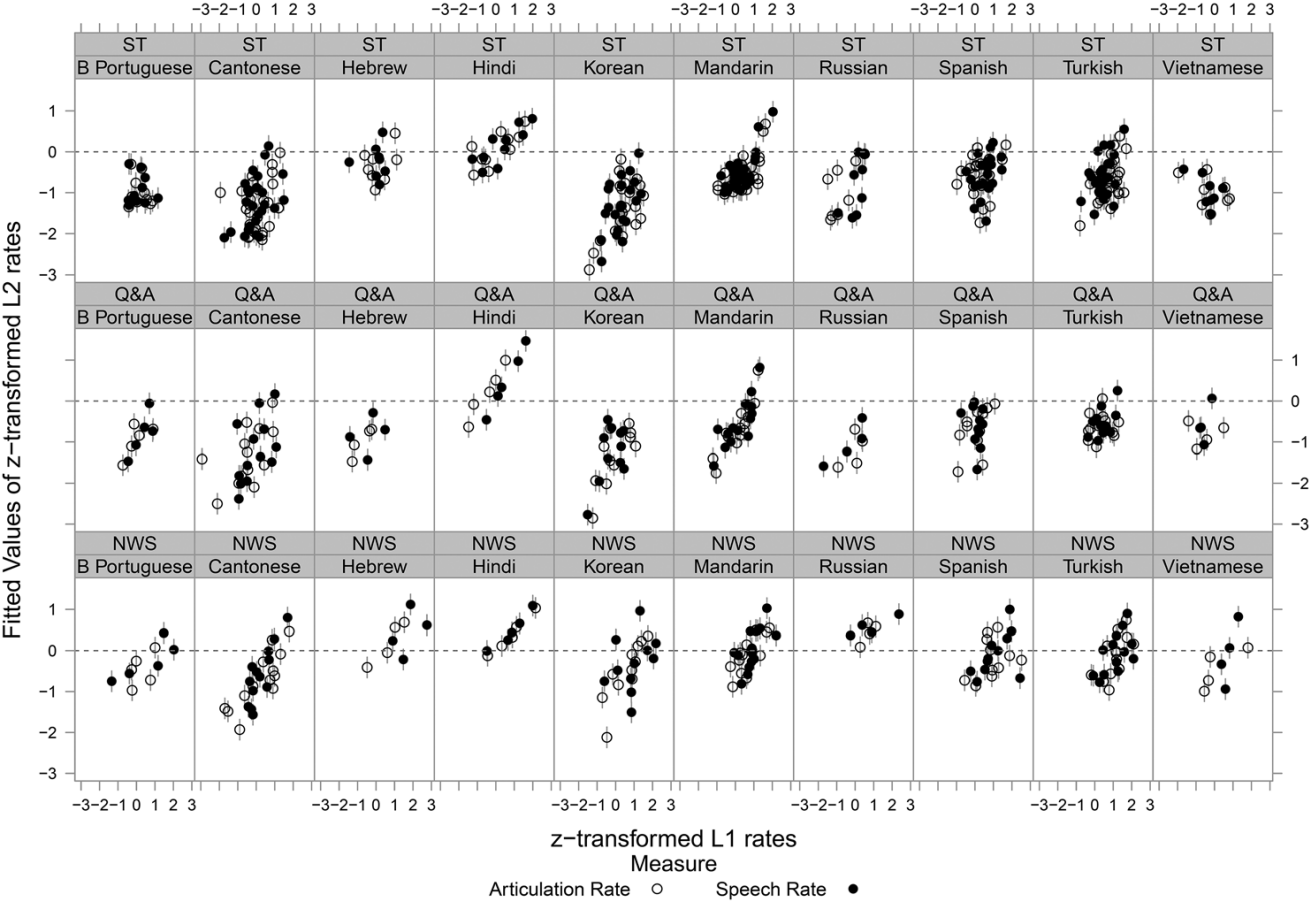
Scatterplots of L1 (various languages) speaking rate (*z*-transformed) vs fitted values of *z*-transformed L2 (English) speaking rate for individual bilingual talkers by task [top row = story narratives (ST), middle row = question prompted narratives (Q&A), bottom row = paragraph reading (NWS)] and by L1.

Finally, in the critical analysis for the present study, we assessed whether individual bilingual talkers' L2 speaking rates could be predicted by their L1 speaking rates (see Fig. 1). (See Appendix D, Table VI, for the estimate, standard error of the estimate, and significance level for all significant fixed effects in this model.) The analysis showed significant main effects of L1 rate (positive relationship between L1 rate and L2 rate), L1 [two languages differed significantly from the grand mean of L2 rates, Hindi (slightly faster) and Korean (slightly slower)], task (read L2 speech was faster than L2 spontaneous speech), measure (L2 articulation rate was slower than L2 speech rate), and both L1 utterance length and L2 utterance length (L1 utterance length negatively, and L2 utterance length positively correlated with L2 speaking rate). While it is unclear why shorter L1 utterances would predict faster L2 speaking rates, the finding that longer L2 utterance lengths predict faster L2 speaking rates parallels the relation observed within L1 speech (as reported above in the second paragraph of Sec. III, and in prior work, e.g., Quené, 2008, and Jacewicz *et al.*, 2010). Talker gender was also included in this analysis, but did not yield a significant main effect. There were significant two-way interactions between task and measure and between L1 and measure. No other interactions were significant. Critically for the present study, this analysis showed that bilingual talkers' L2 speaking rates were robustly predicted by their L1 speaking rates. Moreover, in an analysis that included proficiency as a control variable [i.e., an analysis with only those bilingual talkers (*n* = 58) for whom proficiency (Versant) scores were available], the critical relation between L1 and L2 speaking rates remained. (See Appendix E, Table VII, for the estimate, standard error of the estimate, and significance level for all significant fixed effects in this model.)

**TABLE VI.** Significant fixed effects for the analysis of predictors of L2 speaking rate variation. Significance codes: *** = 0; ** = 0.001; * = 0.01; . = 0.05. Dependent variable was L2 speech rate and articulation rate, *z*-transformed. Random intercept was talker, with task included as the random slope.

	Estimate	Standard Error	df	*t*-value	Pr (>|*t*|)	
(Intercept)	−0.67	0.05	97	−12.75	<2 × 10^−16^	***
Task (NWS vs Q&A + ST)	−0.37	0.11	108	−3.41	0.00	***
Measure	0.18	0.05	502	3.44	0.00	***
L1 speaking rate (*z*-score)	0.35	0.04	436	9.07	<2 × 10^−16^	***
L1 Hindi	0.61	0.16	75	3.74	0.00	***
L1 Korean	−0.39	0.12	75	−3.33	0.00	**
L1 Utterance length	−0.06	0.02	244	−3.70	0.00	***
L2 Utterance length	0.15	0.02	140	8.04	0.00	***
Task (NWS vs Q&A + ST):Measure	0.46	0.17	450	2.72	0.01	**
Task (Q&A vs ST):Measure	0.33	0.09	454	3.73	0.00	***
Task (Q&A vs ST):L1 speaking rate	−0.15	0.07	174	−2.22	0.03	*
Task (NWS vs Q&A + ST):L1 Korean	−0.37	0.18	75	−2.08	0.04	*
Task (NWS vs Q&A + ST):L1 Russian	−0.89	0.27	69	−3.30	0.00	**
Measure:L1 Mandarin	−0.21	0.07	386	−2.85	0.00	**
Measure:L1 Hindi	−0.23	0.11	383	−2.08	0.04	*
Measure:L1 Russian	0.47	0.12	389	3.73	0.00	***
Measure:L1 Spanish	0.16	0.08	380	2.00	0.05	*
Task (NWS vs Q&A + ST):Measure:L1 speaking rate	0.30	0.15	424	2.06	0.04	*

**TABLE VII.** Significant fixed effects for the analysis of predictors of L2 speaking rate variation with proficiency scores controlled. Significance codes: *** = 0; ** = 0.001; * = 0.01. Dependent variable was L2 speech rate and articulation rate, *z*-transformed. Random intercept was talker. Random slopes were task, L2 utterance length, and measure.

	Estimate	Standard Error	df	*t*-value	Pr (>|*t*|)	
(Intercept)	−0.82	0.07	71	−11.77	<2 × 10^−16^	***
Task (NWS vs Q&A + ST)	−0.56	0.16	50	−3.62	0.00	***
Task (Q&A vs ST)	0.24	0.07	68	3.56	0.00	***
L1 speaking rate (z-score)	0.28	0.05	265	5.78	0.00	***
L1 Korean	−0.32	0.13	47	−2.43	0.02	*
L1 Utterance Length	−0.06	0.02	141	−3.76	0.00	***
L2 Utterance Length	0.23	0.02	36	9.35	0.00	***
Task (NWS vs Q&A + ST):Measure	0.54	0.19	247	2.79	0.01	**
Task (Q&A vs ST):Measure	0.45	0.09	235	4.84	0.00	***
Task (Q&A vs ST): L1 speaking rate (z-score)	−0.22	0.08	213	−2.69	0.01	**
Task (Q&A vs ST):L1 Hebrew	0.43	0.20	46	2.17	0.03	*
Task (NWS vs Q&A + ST):L1 Russian	−1.61	0.41	42	−3.89	0.00	***
Task (NWS vs Q&A + ST):L1 Vietnamese	0.81	0.32	43	2.52	0.02	*
Task (Q&A vs ST):L1 Vietnamese	−0.41	0.20	54	−2.11	0.04	*
Task (NWS vs Q&A + ST):Versant	0.03	0.01	44	2.38	0.02	*

## IV. DISCUSSION

In the present study, we asked if some of the observed individual variations in L2 speaking rate could be accounted for by variation across talkers in L1 speaking rate. As discussed in the Introduction, our interest in this question derives from notable developments in experimental and theoretical phonetics that have indicated a close integration of (e.g., Nygaard *et al.*, 1994; Remez *et al.*, 1997; Allen *et al.*, 2003) and listener sensitivity to (e.g., Thompson, 1987; Goggin *et al.*, 1991; Allen and Miller, 2004; Perrachione and Wong, 2007; Winters *et al.*, 2008; Perrachione *et al.*, 2011), indexical and linguistic information in the speech signal. A critical issue raised by this view of speech production and perception is with regard to the language-specificity vs language-generality of indexical characteristics in bilingual talkers. In particular, we asked whether there are any language-independent, talker-specific characteristics that are consistently evident in each of the two languages of a bilingual talker, and that, in combination with the anatomically or physiologically determined features, become part of the unique set of indexical features for that individual regardless of the language being spoken.

We hypothesized that relative speaking rate may be a feature of speech production that reflects some degree of language-independent talker-specific control in language and speech production by bilingual individuals. Our analyses showed first that, at the group level, there were some significant differences in L1 speaking rate across various languages. Specifically, the group of Turkish talkers in our corpus had a faster average speaking rate than the grand mean of the speaking rate of all of the 11 languages included in the study. De Jong *et al.* (2015) also found that L1 Turkish had a faster speaking rate than L1 English speech, and they suggested that this difference may be related to differences in the phonotactics of English and Turkish. Specifically, while English allows complex onsets and codas, fewer consonant clusters are permissible in Turkish. However, inclusion of a wide range of typologically distinct languages in the present study, some of which have even simpler phonotactics (e.g., Mandarin) than Turkish, allowed us to see that relatively complex vs simple phonotactics is an unlikely source on its own of cross-language speaking rate differences. While this difference is noteworthy, identifying the source of this group-level difference remains beyond the scope of the present study (see Pellegrino *et al.*, 2011, for discussion of cross-language rate differences). Instead, for the present focus on individual-level variation, we take these L1 speaking rate measurements as the context in which to assess L2 speaking rate variation, and as points of comparison with the L2 speaking rate measurements both across L1 and L2 talkers of English, as well as within bilinguals across their two languages.

By establishing a dissociation between L1 and L2 speaking rates in absolute terms, with L1 rates being significantly faster than L2 rates (consistent with Guion *et al.*, 2000; Baese-Berk and Morrill, 2015, amongst others), we verified in our corpus that speaking rate is a global speech feature that is systematically influenced by language status as either L1 or L2. Crucially for the goals of the present study, we found a significant positive relationship between L1 and L2 speaking rates within individual bilinguals in relative terms (i.e., individual L1 and L2 rates in relation to the range of L1 and L2 rates across individuals in the group of bilingual talkers). This relationship was observed across bilinguals from ten different native language backgrounds, and was observed regardless of whether L2 proficiency (as assessed by the Versant test) was controlled. Taken together, these results indicate that the general slowing associated with L2 speech production occurs in proportion to an individual's L1 speaking rate. Put another way, overall speaking rate in any given utterance is significantly influenced by both language “state” characteristics (L2-status reliably manifests with a slower speaking rate than L1-status) and talker “trait” characteristics (talkers who are relatively slow in L1 will also generally be relatively slow in L2).

Prior research on the nature of individual variability in L1 speaking rate amongst monolingual talkers has generally focused on physical factors (neuromuscular, anatomical, and physiological, e.g., Tsao and Weismer, 1997; Tsao *et al.*, 2006), linguistic factors (position-in-utterance, utterance length, discourse prominence, etc., e.g., Quené, 2008; Jacewicz *et al.*, 2010; and many others), and group-level factors (dialect and sociolinguistic group, e.g., Jacewicz *et al.*, 2010; Clopper and Smiljanic, 2011; Kendall, 2013, and the numerous references reviewed therein) all of which can contribute to both within- and between-talker variability. By focusing on the L1-L2 speaking rate relationship in bilinguals, the present study documents a level of spoken language control that is both language-independent and talker-specific, but that is also central rather than peripheral (i.e., is not a direct consequence of the size, shape, and function of an individual's vocal tract). That is, in addition to the physical constraints on an individual talker's maximum and habitual speaking rates (see Tsao and Weismer, 1997; Tsao *et al.*, 2006), there are also language-independent speaking rate control mechanisms that contribute to talker-specific variability in bilinguals in both their L1 and L2 (i.e., factors that are neither structural linguistic nor sociolinguistic, nor physical, yet still language-independent and individual-specific). In monolingual talkers, it is difficult to distinguish language-independent from language-specific speaking rate control mechanisms. However, in bilingual talkers we have been able to determine that the general slowing of speaking rate for L2 relative to L1 speech likely occurs within language-independent, individual-specific temporal processing constraints. Consequently, across a group of bilinguals from quite diverse language backgrounds, those talkers who are relatively fast speakers in L1 are generally relatively fast speakers in L2, even though L2 speech is invariably slower than L1 speech within individual bilinguals, and notwithstanding the modulation and constraints imposed by physical, linguistic, and sociolinguistic sources of speaking rate variation. Thus, we can identify two distinct sources of language-independent talker-specificity in speaking rate: physical variations, including neuromuscular, anatomical, and physiological variations, as well as functional linguistic variability, that is, in the speed and/or efficiency of language and speech production. For bilinguals, language-independent talker specificity can therefore emerge quite strongly in the overall temporal structure, or tempo, carried by the speech signal. While the underlying source of this language-independent talker-specificity in speaking rate has not been identified in the present study, we note here the important findings of prior work indicating an influence of a personality trait, extroversion, on fluency of production of both L1 and L2 speech (Dewaele and Furnham, 1999, 2000).

In the present study we examined speaking rate in both read speech and in spontaneous speech with the idea that language-independent talker specificity may be more evident in tasks that involve complex language generation (spontaneous speech) than in a task that emphasizes speech production without language generation at the conceptual level (reading). However, in the present study, the relationship between L1 and L2 speaking rates within bilinguals was similarly evident in both read and spontaneous speech (i.e., there was no task by L1 interaction in the analysis that examined L1 speaking rate as a predictor of L2 speaking rate). Nevertheless, in the analysis of L1 and L2 speaking rate across the various L1s and the analysis that directly compared L1 and L2 speaking rates, the data showed that speaking rate was generally modulated by task such that read speech was produced with a faster speaking rate than spontaneous speech in both L1 and L2 speech production. Moreover, within spontaneous speech, the task with picture prompts elicited speech with a faster rate than the free-form, question-and-answer task. Thus, speaking rate in both L1 and L2 decreased with increasing task complexity. Furthermore, the data showed an interaction between language status (as L1 or L2) and task, such that the L1 vs L2 speaking rate difference was smaller in the simpler task (reading) than in the less constrained spontaneous speech tasks whether picture-guided or question-prompted. Thus, while task differences did not appear to amplify or diminish talker-specificity in speaking rate, the difficulties of L2 speaking seem to accumulate across levels of processing such that L2 speech is particularly slow in complex tasks that require language generation at the semantic and syntactic levels compared to read speech where the talker does not need to generate well-formed phrases and sentences in addition to having to produce intelligible speech (for a similar idea of cumulative effects of reduced efficiency in L2 relative to L1 at all levels of processing, see Cutler *et al.*, 2004).

Language-independent talker-specificity may seem to be of little practical consequence under most circumstances of speech communication since listeners often encounter either the L1 or the L2 speech of an individual bilingual talker without exposure to that bilingual's speech in the other language. However, it is also quite common for bilinguals who share both their L1 and L2 to communicate with each other in both languages depending on the context of a given communication instance. For example, bilinguals who typically communicate in their shared L1 may switch to a shared L2 in a group for which the L2 functions as the lingua franca. Moreover, due to the ubiquitous phenomenon of mid-utterance code switching, bilinguals quite frequently gain familiarity with each other's speech in both languages. It is under these circumstances that the manifestation of language-independent talker-specificity in L1 and L2 speaking rate may contribute to language-independent bilingual talker identification and to language-independent listener adaptation to an individual bilingual talker.

Since L2 speaking rate is consistently slower than L1 speaking rate, it is not possible that listeners develop expectations about the absolute speaking rate of a particular bilingual across the two (or more) languages of that individual talker. How, then, might this aspect of talker-specificity serve as a language-independent idiolectal marker? One possibility is that listeners develop expectations about the range of speaking rates to expect for L1 and L2 speech. Then, when an individual bilingual talker is encountered, and once the L1 or L2 status has been determined on the basis of the wide range of acoustic-phonetic deviations that typically characterize L1 vs L2 speech in a given language (including both spectral and temporal features), listeners could adjust to that individual's speaking rate within the appropriate range. It is then possible that this L1- or L2-specific adjustment could form the basis for generalization to the other of this particular bilingual's language. If a listener has experience with a relatively fast talker in L1, then this listener may expect that this talker will also be a relatively fast talker in L2. This experience-based expectation about speaking rate may then combine with the other talker-specific factors (such as the vocal source characteristics) that facilitate talker identification and recognition of that talker's speech in either the L1 or the L2.

It is also possible that talker-specific, rate-based expectations that listeners develop on the basis of exposure to one language (either L1 or L2 of a given bilingual) may extend to other rate-dependent acoustic-phonetic features that can quite easily generalize across languages. For example, even for an L1-L2 pair with different duration-based phonological contrasts (e.g., a long-short vowel contrast in L1 and a tense-lax vowel contrast in L2), experience with a given talker's realization of the L1 contrast in relation to the L1 norms could potentially provide the basis for expected durations in this talker's realization of an analogous L2 contrast. Moreover, since fast and slow speech are associated with general hypo- and hyper-articulation, respectively, it may be possible for listeners to develop quite extensive, language-independent, talker-specific, rate-based expectations for L1 (or L2) speech on the basis of prior experience with L2 (or L1) speech. Such expectations could then facilitate recognition of this talker's speech across both languages even if prior exposure has been to only one of the talker's languages.

In conclusion, our analyses of speaking rate in L1 and L2 within bilingual talkers has suggested that a significant portion of variation in L2 speech is derived from talker-specific variation in L1 speech. Notwithstanding the strong influence of the relationship between first- and second-language structure, and the similarly strong influence of experience- and training-related second-language proficiency, individual variability in L2 spoken language production is probably best understood within the context of individual variability in L1 spoken language production.

## Footnotes

^1^ http://groups.linguistics.northwestern.edu/speech_comm_group/allsstar/ (Last viewed 2/9/2017).

^2^ https://oscaar.ci.northwestern.edu/ (Last viewed 2/9/2017).

^3^ TOEFL^®^: Test of English as a Foreign Language (Educational Testing Service, ETS); SPEAK: The Speaking Proficiency English Assessment Kit (Educational Testing Service, ETS); The Versant™ English Test: Automatic evaluation of the spoken English skills of non-native English speakers (Pearson Education, Inc., Menlo Park, CA).

^4^ A noteworthy difference between the present study and prior work that established a positive relationship between utterance length and speaking rate (Quené, 2008; Jacewicz *et al.*, 2010) is that, while the present study entered mean utterance length as a predictor in the analyses, the prior work examined the rate-length relationship at the level of individual utterances.

^5^ In contrast to multivariable models, in which the effect of more than one predictor variable is assessed while adjusting for potential confounds from other predictors (i.e., the model includes multiple independent variables), multivariate modeling techniques are designed to assess the influence of predictor variables on multiple outcome measures (i.e., the model includes multiple dependent variables as well as multiple independent variables). Common applications of multivariate regression are longitudinal studies, in which multiple measures are taken at various time points from each participant, and studies with nested or clustered data with multiple participants in each cluster (see Faraway, 2005; Hidalgo and Goodman, 2013). In the present study, the articulation rate measure is nested within the speech rate measure. By providing simultaneous tests for regressions with both dependent variables, the multivariate regression model takes into account this correlation between the two measures, speech rate and articulation rate.
